# Ventilator-Associated Pneumomediastinum With Secondary Pneumothorax in Myasthenic Crisis

**DOI:** 10.7759/cureus.109354

**Published:** 2026-05-21

**Authors:** Nikhila P Archakam, Marissa Oller-Cramsie, Rahul Mittal

**Affiliations:** 1 Medical School, Rowan-Virtua School of Osteopathic Medicine, Stratford, USA; 2 Neurology, Holy Name Medical Center, Teaneck, USA; 3 Health Informatics, Rutgers University, Piscataway, USA

**Keywords:** macklin phenomenon, mechanism of pneumomediastinum, myasthenia gravis crisis, myasthenia gravis (mg), prolonged mechanical ventilation, pulmonary barotrauma, secondary pneumothorax, subcutaneous emphysema

## Abstract

Myasthenia gravis (MG) is an autoimmune disorder of the neuromuscular junction that can progress to myasthenic crisis, a life-threatening condition requiring mechanical ventilation. While ventilatory support is essential, it carries the risk of barotrauma, including pneumomediastinum and pneumothorax. We present the case of a 58-year-old female with a history of MG who developed acute hypercapnic respiratory failure requiring intubation and mechanical ventilation. Her clinical course was complicated by pneumomediastinum with associated subcutaneous emphysema and a secondary pneumothorax identified on CT imaging following a prolonged intensive care stay. The pathophysiology of ventilator-associated barotrauma involves alveolar overdistension and rupture, allowing air to track along bronchovascular sheaths into the mediastinum (Macklin effect), with potential extension into the pleural space and subcutaneous tissues. In this case, contributing factors likely included prolonged mechanical ventilation, underlying bronchiectasis with paraseptal bullous changes, and immunosuppression. The patient was managed conservatively with close monitoring and multidisciplinary care, resulting in clinical stability without the need for surgical intervention. This case highlights a rare but clinically significant complication of mechanical ventilation in myasthenic crisis and underscores the importance of early recognition of barotrauma. Awareness of this potential complication is essential for timely diagnosis and appropriate management in critically ill patients with MG.

## Introduction

Myasthenia gravis (MG) is an autoimmune disorder of the neuromuscular junction characterized by fluctuating, fatigable muscle weakness. Myasthenic crisis is defined as respiratory muscle weakness leading to respiratory failure requiring mechanical ventilation and occurs in approximately 10-20% of patients during the disease course [1]. The decision to intubate in myasthenic crisis is guided by serial bedside respiratory assessments, including forced vital capacity (FVC), negative inspiratory force (NIF), and maximal expiratory pressure (MEP). Commonly referenced thresholds suggesting impending respiratory failure include FVC below 20 mL/kg, NIF worse than -30 cmH₂O, and MEP less than 40 cmH₂O, particularly when accompanied by progressive hypercarbia and clinical signs of increased work of breathing such as tachypnea, accessory muscle use, and paradoxical abdominal breathing [2]. Additional risk factors for myasthenic crisis include prior crises, severe untreated disease, bulbar involvement, thymoma, and chronic immunosuppression.

Patients with MG are particularly susceptible to pulmonary complications. Respiratory muscle weakness impairs effective cough and secretion clearance, predisposing patients to aspiration and recurrent lower respiratory tract infections, while immunosuppressive therapies further increase infection risk [3]. These factors may both precipitate and complicate myasthenic crises.

Pneumomediastinum may arise from a variety of etiologies beyond mechanical ventilation. Spontaneous pneumomediastinum has been associated with severe coughing, asthma exacerbations, retching, and Valsalva maneuvers, while secondary causes include esophageal perforation, tracheobronchial injury, and positive-pressure ventilation [4-6]. In critically ill patients, iatrogenic causes such as endotracheal intubation and bronchoscopy must also be considered. Therefore, a broad differential diagnosis is essential when pneumomediastinum is identified to avoid premature attribution to a single etiology.

Mechanical ventilation is often life-saving in myasthenic crisis; however, it carries the risk of barotrauma. Alveolar overdistension is the primary mechanism of injury and may result in pneumomediastinum, which can progress to pneumothorax in up to 42% of affected cases [4]. Although barotrauma occurs in only a minority of mechanically ventilated patients, the risk increases in the presence of elevated airway pressures, prolonged duration of ventilation, high tidal volumes, and preexisting structural lung disease such as emphysema, bullae, or bronchiectasis [4]. Lung-protective ventilation strategies using low tidal volumes and limiting plateau pressures have been shown to reduce this risk. The pathophysiology is classically mediated by alveolar rupture with air tracking along bronchovascular sheaths into the mediastinum, known as the Macklin effect [5].

Patients with MG may be uniquely predisposed to ventilator-associated barotrauma due to several disease-specific factors. Chronic respiratory muscle weakness contributes to recurrent pulmonary infections and impaired secretion clearance, which, over time, may lead to structural lung changes, including bronchiectasis. In addition, chronic immunosuppressive therapy, including corticosteroids, mycophenolate mofetil, and newer biologic agents such as FcRn inhibitors, may further increase susceptibility to infection and progressive parenchymal injury. MG-associated respiratory failure frequently requires prolonged mechanical ventilation while immunomodulatory therapies take effect and respiratory muscle strength recovers, increasing cumulative alveolar stress [7]. Patients with concomitant pulmonary conditions, including nontuberculous mycobacterial infections, may develop additional structural abnormalities such as paraseptal bullous disease, further compromising alveolar integrity and lowering the threshold for alveolar rupture. These overlapping risk factors may create a synergistic vulnerability to barotrauma that distinguishes MG patients from the general mechanically ventilated population. We present a case of ventilator-associated pneumomediastinum with secondary pneumothorax occurring in the setting of myasthenic crisis, prolonged mechanical ventilation, chronic immunosuppression, and underlying structural lung disease.

## Case presentation

A 58-year-old female with a relevant history of MG status post-thymectomy and radiation therapy (2016), prior myasthenic crises requiring intubation and intravenous immunoglobulin therapy (last episode in 2017), cholangiocarcinoma status post-cholecystectomy with associated paraneoplastic autoantibodies, and pulmonary embolism on apixaban presented on November 28, 2025, with progressive dyspnea, cough, and generalized weakness.

Her history was notable for prior sputum cultures positive for *Mycobacterium* *abscessus*, for which she received guideline-directed multidrug therapy per American Thoracic Society/Infectious Diseases Society of America recommendations, including a macrolide, amikacin, and a parenteral beta-lactam. Chronic *M. abscessus* infection likely contributed to the development of bronchiectasis and paraseptal bullous changes identified on subsequent imaging, representing structural lung disease that may have increased susceptibility to alveolar rupture during mechanical ventilation.

On presentation, she was tachycardic. Laboratory evaluation demonstrated mild transaminitis, and respiratory viral panel testing was negative. Arterial blood gas analysis (ABG) demonstrated hypoxia with chronic compensated respiratory acidosis, with elevated pCO₂ of 59 mmHg and compensatory bicarbonate of 34.9 mmol/L. Initial laboratory evaluation is summarized in Table 1.

**Table 1:** Laboratory values on admission.

Parameter	Value	Units	Reference range
pH	7.38	-	7.35–7.45
pCO₂	59 ↑	mmHg	35–45
pO₂	76 ↓	mmHg	80–100
HCO₃⁻	34.9 ↑	mmol/L	22–26
O₂ saturation	94.8 ↓	%	95–100
Base excess	+7.7 ↑	mmol/L	0–2
Glucose	125 ↑	mg/dL	70–99
Sodium	138	mmol/L	135–145
Potassium	4.6	mmol/L	3.5–5.2
Chloride	97 ↓	mmol/L	98–108
CO₂ (serum)	30	mmol/L	22–30
Blood urea nitrogen	10	mg/dL	10–20
Creatinine	0.47 ↓	mg/dL	0.55–1.02
Calcium	8.9	mg/dL	8.4–10.3
Aspartate aminotransferase	62 ↑	U/L	6–55
Alanine aminotransferase	38 ↑	U/L	5–34
Alkaline phosphatase	103	U/L	40–150
White blood cell count	10.7 ↑	K/µL	4.0–10.5
Hemoglobin	12.5	g/dL	12.0–15.7
Platelets	192	K/µL	140–450

CT of the chest on November 29, 2025, demonstrated bronchiectasis with mucus plugging in the right lower lobe and scattered opacities concerning for infection. The presence of bronchiectasis is clinically significant in this context, as it reflects chronic structural airway damage likely resulting from recurrent lower respiratory tract infections in the setting of immunosuppression and impaired secretion clearance due to respiratory muscle weakness. These structural changes reduce alveolar integrity and may lower the threshold for alveolar rupture during positive-pressure ventilation, thereby increasing susceptibility to barotrauma.

Her respiratory status deteriorated during hospitalization, with increasing tachypnea and oxygen saturation in the low 90s despite high-flow oxygen. Serial bedside pulmonary function testing, including FVC and NIF, guided the decision to proceed with intubation, consistent with the 20-30-40 rule. She was transferred to the intensive care unit (ICU) and required endotracheal intubation and mechanical ventilation. ABG at that time showed a pH of 7.25, pCO₂ of 68 mmHg, and pO₂ of 162 mmHg, consistent with acute hypercapnic respiratory failure. Repeat chest radiography demonstrated bilateral infiltrates.

Mechanical ventilation was initiated using pressure-regulated volume control (PRVC) mode with a tidal volume of approximately 450 mL (9.4 mL/kg ideal body weight) and positive end-expiratory pressure (PEEP) of 5 cmH₂O. Peak and plateau pressures were not consistently documented and were unavailable for review.

She was treated with intravenous cefepime for presumed pneumonia and received immunomodulatory therapy for myasthenic crisis, including intravenous immunoglobulin (Privigen 10%) administered at a dose of 40 g daily over multiple days, along with corticosteroid therapy using methylprednisolone (40 mg per dose). She remained on mycophenolate mofetil, titrated to a maximum dose of 1,000 mg daily, for chronic immunosuppression. During her ICU course, she required transient vasopressor support, reflecting the severity of her critical illness. Antimicrobial therapy included fluconazole (200 mg daily), administered empirically given the patient’s prolonged critical illness and immunosuppressed state, which conferred an elevated risk for invasive fungal infection. Supportive care included bronchodilator therapy with ipratropium-albuterol nebulizations, as well as sedation and analgesia with midazolam and morphine. Gastrointestinal prophylaxis was provided with pantoprazole.

Bronchoscopy revealed a normal tracheobronchial tree with intact mucosa and minimal secretions, without endobronchial lesions or mucosal abnormalities. During hospitalization, she developed fever (101.7°F) and leukocytosis with neutrophilic predominance. She remained on pyridostigmine, prednisone, and supportive care. After a prolonged ICU course requiring 14 days of mechanical ventilation, she was successfully weaned and discharged in stable condition.

The patient was readmitted approximately three months after her initial hospitalization, in February 2026. CT imaging on February 10, 2026, demonstrated pneumomediastinum with associated subcutaneous emphysema and a small posterior right apical pneumothorax (Figure 1). Additional findings included paraseptal bullous changes in the right upper lobe. The patient’s clinical course is summarized in Figure 2.

**Figure 1:**
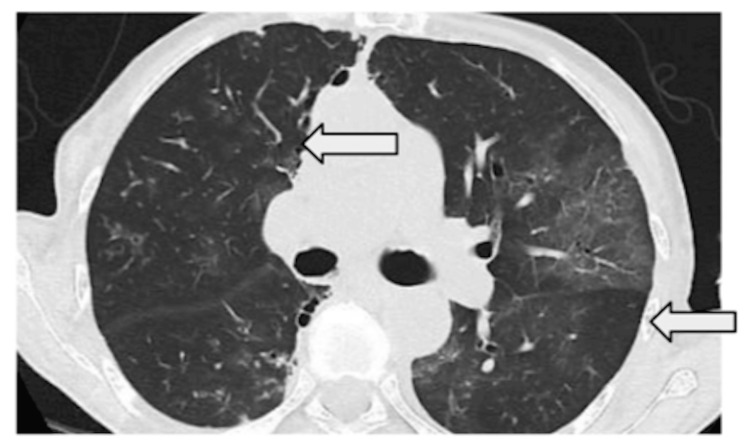
Axial CT of the chest (lung window) demonstrating pneumomediastinum (arrows) with air outlining mediastinal structures and associated subcutaneous emphysema within the chest wall soft tissues, consistent with ventilator-associated barotrauma.

**Figure 2:**
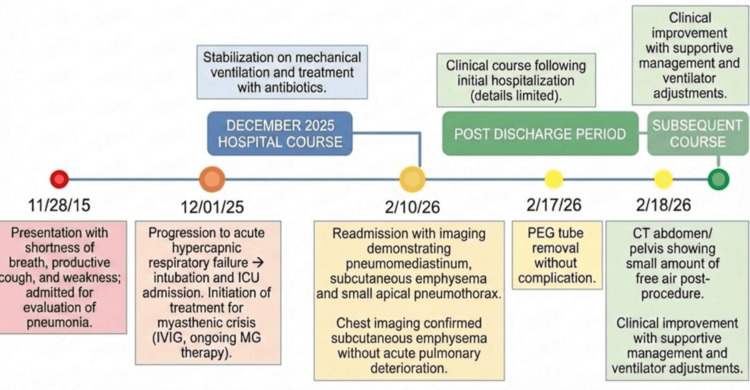
Clinical timeline of myasthenic crisis and subsequent ventilator-associated pneumomediastinum and pneumothorax. Clinical timeline of the patient’s course from initial presentation in November 2025 with myasthenic crisis requiring intubation and mechanical ventilation, to subsequent readmission in February 2026 with pneumomediastinum and pneumothorax consistent with ventilator-associated barotrauma (Macklin effect). Created using Microsoft PowerPoint. ICU: intensive care unit; IVIG: intravenous immunoglobulin; MG: myasthenia gravis; PEG: percutaneous endoscopic gastrostomy

A previously placed percutaneous endoscopic gastrostomy tube was removed, and nutritional status was optimized with oral supplementation. Multidisciplinary care involving neurology, pulmonology, gastroenterology, infectious disease, and thoracic surgery was provided. Conservative management of the pneumomediastinum and pneumothorax was recommended without intervention. MG therapy was continued with adjustments to pyridostigmine, mycophenolate mofetil, and prednisone. The patient remained clinically stable before discharge.

## Discussion

This case highlights a rare complication of prolonged mechanical ventilation in myasthenic crisis resulting in pneumomediastinum and pneumothorax, which has been described in mechanically ventilated patients with underlying lung pathology [4,6]. Barotrauma in mechanically ventilated patients is classically associated with elevated airway pressures, including increased peak inspiratory pressure and PEEP, which can lead to alveolar overdistension and rupture. In this case, the patient required prolonged mechanical ventilation, which increases cumulative exposure to ventilator-associated lung injury and the risk of barotrauma [4]. Available ventilator settings included PRVC mode with tidal volume of approximately 450 mL (approximately 9 mL/kg predicted body weight), exceeding the ARDSNet lung-protective threshold of 6-8 mL/kg, and PEEP of 5 cmH₂O. This suggests that cumulative exposure to relatively elevated tidal volumes may have contributed to barotrauma risk in this patient, even in the absence of overtly elevated airway pressures.

Alveolar rupture allows air to dissect along bronchovascular sheaths toward the mediastinum, a process known as the Macklin effect. A schematic representation of the Macklin effect is shown in Figure 3.

**Figure 3:**
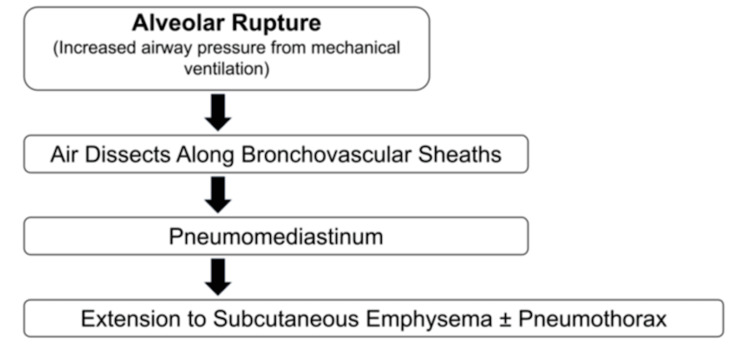
Schematic representation of the Macklin effect. Schematic representation of the Macklin effect demonstrating alveolar rupture with air tracking along bronchovascular sheaths into the mediastinum, resulting in pneumomediastinum with potential extension into subcutaneous tissues and the pleural space. Created using Microsoft PowerPoint.

This mechanism explains the development of pneumomediastinum, which may progress to subcutaneous emphysema and pneumothorax, as observed in this patient. It is important to acknowledge that pneumomediastinum may also develop in the absence of mechanical ventilation, particularly in the setting of severe cough or forceful exhalation against a closed glottis, generating high intrathoracic pressures sufficient to cause alveolar rupture. In this patient, the development of pneumomediastinum during a subsequent hospitalization following discharge makes it difficult to definitively attribute the finding solely to mechanical ventilation. However, given her underlying bronchiectasis, paraseptal bullous changes, and history of prolonged mechanical ventilation, barotrauma remains the most likely contributing mechanism. Therefore, this case highlights the importance of considering both ventilator-associated and spontaneous causes of pneumomediastinum in patients with myasthenic crisis and structural lung disease.

Additionally, this case illustrates the management challenges of severe MG in a patient receiving Fc receptor (FcRn) inhibitor therapy and multiple immunosuppressive agents. Several factors contributed to the complexity of this patient’s clinical course.

First, the patient experienced severe disease requiring mechanical ventilation despite ongoing immunotherapy with the FcRn inhibitor rozanolixizumab. Patients with myasthenic crisis requiring mechanical ventilation have been shown to experience significant morbidity and prolonged ICU courses. Rozanolixizumab (Rystiggo), a neonatal FcRn inhibitor, reduces circulating IgG levels by blocking FcRn-mediated recycling. While effective in managing generalized MG, this mechanism may increase susceptibility to infection by reducing protective IgG antibody levels.

In this patient, immunosuppression with both rozanolixizumab and mycophenolate mofetil may have contributed to recurrent infections and a prolonged ICU course, indirectly increasing the risk of barotrauma. Mycophenolate mofetil, a purine synthesis inhibitor that suppresses T and B-lymphocyte proliferation, further impairs humoral and cellular immunity, compounding the infection risk associated with FcRn inhibitor therapy. Notably, mycophenolate mofetil was continued during the acute hospitalization despite suspected pneumonia. While immunosuppressive agents theoretically increase infection susceptibility, abrupt discontinuation in MG carries a significant risk of disease exacerbation and worsening myasthenic crisis. The decision to continue mycophenolate reflected the clinical judgment that the risks of MG exacerbation outweighed the risks of continued immunosuppression, and was made in coordination with the neurology and infectious disease teams, with close monitoring for worsening infection.

Second, the patient developed pneumomediastinum and pneumothorax during a subsequent hospitalization, which likely represents delayed barotrauma following prolonged mechanical ventilation [6]. However, alternative mechanisms must also be considered. Pneumomediastinum can develop in the setting of forceful coughing against a closed glottis, which may occur independently of mechanical ventilation, particularly in patients with underlying respiratory muscle dysfunction and chronic cough from bronchiectasis. While the temporal relationship to the preceding prolonged ventilatory course and the presence of structural lung disease favor ventilator-associated barotrauma as the primary etiology, a contribution from non-ventilator-related mechanisms cannot be excluded.

Third, the patient’s underlying bronchiectasis and paraseptal bullous changes likely increased susceptibility to alveolar rupture and air leak. Pneumomediastinum has been associated with both positive-pressure ventilation and preexisting lung pathology, which together may significantly increase the risk of barotrauma [6]. Risk factors contributing to barotrauma in this patient are summarized in Table 2.

**Table 2:** Risk factors for barotrauma in myasthenia gravis. Risk factors for barotrauma in myasthenia gravis, including patient-specific and disease-related contributors.

Risk factor	Mechanism	Present in this case
Prolonged mechanical ventilation	Cumulative alveolar stress and overdistension	Yes
Positive-pressure ventilation	Increased transalveolar pressure leading to rupture	Yes
Underlying structural lung disease (bronchiectasis, bullae)	Reduced alveolar integrity, predisposition to air leak	Yes
Respiratory muscle weakness (myasthenia gravis)	Impaired ventilation → prolonged ventilatory support	Yes
Immunosupression	Increased infection risk → prolonged intensive care unit course	Yes
Recurrent infections	Inflammation and lung injury increasing susceptibility to barotrauma	Yes

Additionally, the patient experienced severe protein-calorie malnutrition requiring enteral nutritional support during hospitalization, further complicating recovery. Immunosuppression further increased the risk of infectious complications and contributed to overall clinical complexity.

Despite these challenges, the patient was successfully weaned from mechanical ventilation and later remained clinically stable with conservative management of pneumomediastinum and pneumothorax. Pneumomediastinum is often managed conservatively in stable patients, with resolution occurring without invasive intervention [6]. This case demonstrates that favorable outcomes can still be achieved in complex patients with severe MG when managed with coordinated multidisciplinary care [7,8].

Limitations

This case report has several limitations. As a single-patient observation, its findings are not generalizable and cannot establish causality. Additionally, the precise timing of alveolar rupture could not be determined during the patient’s prolonged ICU course, limiting the ability to definitively link specific ventilator parameters to the development of pneumomediastinum and pneumothorax. Furthermore, the incomplete availability of detailed ventilator settings over the entire hospitalization restricts a more comprehensive analysis of barotrauma risk factors. Specifically, peak inspiratory and plateau pressures were unavailable, limiting full characterization of barotrauma risk attributable to ventilator pressures. Additionally, optimal CT imaging slices most clearly demonstrating the extent of pneumomediastinum were not available for inclusion, which may limit radiographic clarity of this finding. Despite these limitations, this case provides clinically relevant insight into a rare complication of mechanical ventilation in patients with MG.

## Conclusions

This case highlights the complex management of myasthenic crisis in a patient with multiple comorbidities and chronic immunosuppression. The patient developed ventilator-associated pneumomediastinum and pneumothorax during prolonged mechanical ventilation, likely related to barotrauma in the setting of underlying bronchiectasis and paraseptal bullous changes. Additional challenges included *Mycobacterium* abscessus colonization, severe protein-calorie malnutrition, and gastrointestinal complications during hospitalization. Despite these factors, the patient was successfully managed with intravenous immunoglobulin therapy, ventilatory support, and multidisciplinary care. This case underscores the importance of close monitoring for ventilator-associated complications and coordinated management in patients with severe MG. Early recognition of barotrauma in mechanically ventilated patients with myasthenic crisis is critical to prevent progression and guide appropriate management.
